# Beneficial Effects of Caraway Oil in Aluminium Chloride-Induced Neurotoxicity

**DOI:** 10.7759/cureus.86783

**Published:** 2025-06-26

**Authors:** Sandip T Auti, Yogesh A Kulkarni

**Affiliations:** 1 Shobhaben Paratapbhai Patel School of Pharmacy and Technology Management, SVKM's Narsee Monjee Institute of Management Studies (NMIMS), Deemed to be University, Mumbai, IND

**Keywords:** acetylcholinesterase, aluminium chloride, alzheimer's disease, caraway oil, neuroprotective

## Abstract

Purpose: Alzheimer's disease (AD) is characterized by cognitive decline and memory impairment, amyloid plaques, and neurofibrillary tangles (NFT). Current therapies provide symptomatic treatment but do not address the exact cause of the disease. Caraway oil, derived from *Carum carvi*, is rich in carvone and limonene with reported anticholinesterase, antioxidant, and neuroprotective properties. This study aimed to evaluate the neuroprotective effect of caraway oil in an aluminum chloride-induced rat model of neurotoxicity.

Methods: Albino Wistar rats were randomized into five groups: normal control, disease control (aluminum chloride, 100 mg/kg), standard (donepezil, 1 mg/kg), and caraway oil treatment groups (100 and 200 mg/kg). Treatments were administered orally for 42 days. Behavioral assessments included locomotor activity, the Morris water maze, the elevated plus maze, and passive avoidance tests. Acetylcholinesterase (AChE) activity and oxidative stress markers were assessed in the hippocampus and cortex.

Results: Caraway oil administration significantly improved locomotor activity and spatial memory in rats at 100 mg/kg and 200 mg/kg. The oil showed a significant effect on oxidative stress parameters in the hippocampus and cortex. AChE activity was also improved significantly (p<0.001) after caraway oil treatment.

Conclusion: Caraway oil demonstrated significant neuroprotective effects in aluminum chloride-induced neurotoxicity, improving cognitive and behavioral functions and reducing oxidative stress. These findings suggest that caraway oil may have therapeutic potential in the management of AD.

## Introduction

Alzheimer’s disease (AD) is the most common cause of dementia and cognitive impairments in older people (aged ≥ 65 years) throughout the world, comprising 60% to 80% of cases worldwide [1]. AD is caused by structural and functional damage to the central nervous system (CNS), which includes neurodegenerative processes and aberrant nervous system protein aggregation [2]. AD has been linked to two different kinds of lesions: neurofibrillary tangles (NFT), which form in neurons as a result of the hyperphosphorylated tau protein, and amyloid plaques, which are made of beta-amyloid peptides (Aβ) and accumulate abnormally outside of nerve cells [3]. AD can be considered a progressive process of biochemical, neurophysiological, and neuroanatomical changes. [4]. Aluminum is reported to have neurotoxic and cholinotoxic effects; it promotes the accumulation and aggregation of amyloid beta and tau protein in the brain [5]. It has been reported that exposure to aluminum activates the acetylcholinesterase (AChE) enzyme and increases lipid peroxidation in different brain regions of rodents [6].

Although numerous therapeutic approaches have been investigated in clinical trials, the majority of the medications that are currently in the market are symptom-management measures rather than true cure. As a result, the focus has shifted to preventing or lowering the risk of AD [7]. Memantine and cholinesterase inhibitors are used to enhance behavior and cognitive performance; however, they are not able to treat the root cause of brain damage. Furthermore, aducanumab and lecanemab, two anti-amyloid antibodies, have demonstrated promising results in removing amyloid plaques, and research is still ongoing to determine their long-term effects and any adverse effects [8]. Several essential oils have been reported to be used in the management of neurodegenerative diseases [9]. Caraway oil is obtained from the dried ripe fruits of the plant *Carum carvi* (Apiaceae), which is also known as "jeera" in Hindi. The major constituent of the oil is carvone (45% to 65%), with other minor constituents such as limonene, dihydro-carvone, borneol, terpinene, β-pinene, and starch. Its main components are volatile oil (2.5%-8%), fixed oil (10%), proteins (15%), and resins [10]. Caraway oil has many biological properties, such as anticholinesterase, anticonvulsant, and antioxidant properties [11]. It has been demonstrated that carvone inhibits the acetylcholinesterase enzyme and acts as an anti-inflammatory [12]. Based on these findings, the present study was designed to evaluate the neuroprotective effect of caraway oil in aluminum chloride-induced cognitive impairments and oxidative damage in rats.

## Materials and methods

Materials

The details of the procurement of materials are listed in Table 1.

**Table 1:** Details of the materials procured for the studies

Sr. No	Material	Company
1.	Aluminium chloride	Sigma Aldrich (St. Louis, MO, USA)
2.	Acetylthiocholine iodide	Sigma Aldrich (St. Louis, MO, USA)
3.	Caraway oil	iFRAGRANCE INDIA (Kannauj, Uttar Pradesh, India)
4.	Donepezil hydrochloride	Micro Labs Limited (Mumbai, Maharashtra, India)

All other chemicals used were of analytical grade.

Methods

Volatile Oil Analysis

Attenuated total reflectance-Fourier transform infrared spectroscopy (ATR-FTIR): The ATR-FTIR spectrum of the caraway oil was obtained using IR correlation charts. The liquid film of the sample was placed over the quartz crystal surface, and the IR spectra were reported in % transmittance. The wave number region for the analysis was kept at 4000-400 cm⁻¹ on a spectrum equipped with an attenuated total reflectance device and a deuterated triglycine sulfate (DTGS) detector [13].

Nuclear Magnetic Resonance (NMR) Analysis

1H NMR of caraway oil was analyzed by an NMR spectrometer using an ECZR Series 600 MHz system (JEOL Ltd., Tokyo, Japan). The stock solution of the sample was prepared in deuterated chloroform (CDCl3) and kept in NMR tubes; the resulting solutions were sonicated before recording the spectra and capped with a Teflon septum [14].

Gas Chromatography (GC)-Mass Spectrometry (MS) Analysis

An Agilent 6890 N Network GC system (Agilent Technologies, Inc., Santa Clara, CA) was used for the analysis of the sample. The parameters that were set during analysis are listed in Table 2.

**Table 2:** Gas chromatography-mass spectrometry (GC-MS) parameters

Sr. No	Components	Details
1.	Capillary column	BPX 35, 30 m × 0.25 mm; film thickness 0.25 μm (coated with 35% phenyl polysilphenylene-siloxane)
2.	Carrier gas	Helium (1.0 ml/min)
3.	Programme temperature	50°C to 220°C at a rate of 10°C per minute
4.	Split ratio	1:25
5.	Detector	Flame ionization
6.	Acquisition mass range	40–400 amu

A 2% solution of caraway oil was prepared in ethanol (95%), and two replicates of the samples were processed in the same way. The injection volume was kept at 1.0 µl. The identification of the compounds from the caraway oil was done and compared concerning their retention times and mass spectra obtained from authentic Wiley libraries (available through Hewlett-Packard, Palo Alto, CA, USA) and the literature [15]. 

Experimental Animals

Male albino Wistar rats (180-250 g) were procured from the National Institute of Biosciences, Pune, India. Animals were kept at a 12-hour light and 12-hour dark cycle at a room temperature of 22 ± 2℃. Animals were acclimatized for seven days before the experiment. The animals were provided with a sufficient amount of water and a standard pellet diet. The study protocol was approved by the Shri Vile Parle Kelavani Mandal's (SVKM) Institutional Animal Ethics Committee (approval no. CPCSEA/IAEC/P-57/2017).

Experimental Design

According to body weight, experimental animals were randomized into five groups. Each group had 12 animals. Neurotoxicity was induced in male albino Wistar rats with the administration of aluminum chloride (100 mg/kg, p.o.) [16]. Group I received distilled water. Aluminum chloride solution was prepared in distilled water freshly and administered to Groups II, III, IV, and V. Group III animals received standard treatment (donepezil hydrochloride 1 mg/kg p.o.) for 42 days along with aluminum chloride. Groups IV and V received caraway oil at 100 and 200 mg/kg per oral (p.o.), respectively, for 42 days. Treatment was administered one hour after aluminum chloride administration.

Behavioural Parameters

The locomotor activity, spatial memory, and anxiety-like behavior were assessed in rodents using different behavioral tests. The locomotor activity was evaluated by using the actophotometer [16]. The spatial memory and learning were evaluated by the Morris water maze test [17] and the passive avoidance test [18]. For the evaluation of anxiety-like behavior in rodents, an elevated plus maze test was performed [19]. These parameters were done on days 21 and 42.

Biochemical evaluation

Collection of Brain Tissue and Preparation of Tissue Homogenate

All of the animals were sacrificed by carbon dioxide (CO₂) asphyxiation after the behavioral assessment. After brain tissue was collected, the cortex and hippocampus regions were separated and kept at -80℃ and utilized to prepare brain homogenate. The tissue of the cortex and hippocampal regions was homogenized using a probe homogenizer (Polytron PT 2500E, Kinematica, Malters, Switzerland) in 10 volumes of ice-cold 0.1M phosphate buffer solution (pH 7.4). To prevent a temperature rise, the tubes were placed in ice. The post-nuclear supernatant was prepared by centrifuging the homogenate at 2500 g for 20 minutes at 4°C. To obtain the post-mitochondrial supernatant, the entire homogenate was centrifuged at 10,000 g for 20 minutes at 4°C [20].

AChE Activity

The level of AChE in brain homogenate was estimated using the method described in published literature [21].

Oxidative Stress Parameters

The cortex and hippocampus parts of the brain were homogenized in 10 volumes of ice-cold 0.1M phosphate buffer solution (pH 7.4) using a probe homogenizer. Malondialdehyde level was determined by the Ohkawa et al. method in the hippocampus and cortex [22]. Superoxide dismutase assay was measured in the post-mitochondrial supernatant [23]. Post-nuclear supernatant was used to perform the catalase assay [24]. Ellman's method was used to calculate reduced glutathione levels in the hippocampus and cortex [25].

Statistical analysis

All the data were expressed as mean ± standard error of the mean (SEM). Statistical analysis was done by using GraphPad Prism 8 software (GraphPad Software, La Jolla, CA). One-way ANOVA, followed by Dunnett's multiple comparison test, was used for analysis. p<0.05 was kept as the level of significance.

## Results

ATR-FTIR analysis of caraway oil

**Figure 1:**
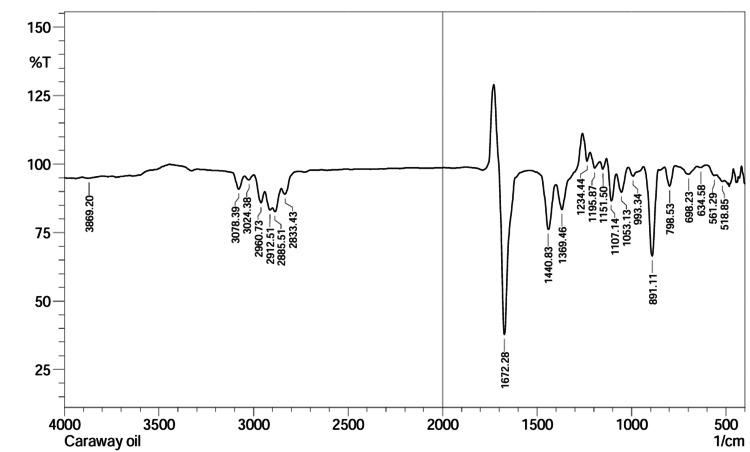
Infrared spectra of caraway oil

In ATR-FTIR spectra, caraway oil showed the presence of functional groups at 3078 cm⁻¹ (C-H str), 2960-2970 cm⁻¹ (Ar C-H aldehydic), 1672 cm⁻¹ (C=C alkene), and 1515 cm⁻¹ (Ar C=C str) (Figure 1).

NMR analysis of caraway oil

**Figure 2:**
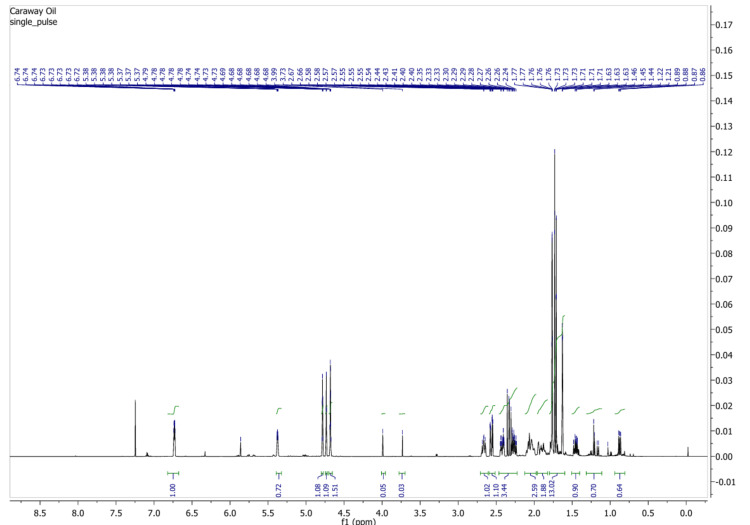
Nuclear magnetic resonance (NMR) spectra of caraway oil

NMR spectra of caraway oil showed 1H-NMR (400 MHz) δ 12.92 (s, 1H), 10.89 (s, 1H), and 8.36 (s, 1H) (Figure 2).

GC-MS analysis of caraway oil

**Figure 3:**
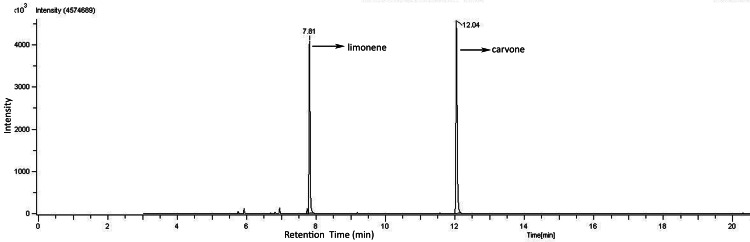
Gas liquid chromatography (GLC) fingerprint profile of caraway oil

**Figure 4:**
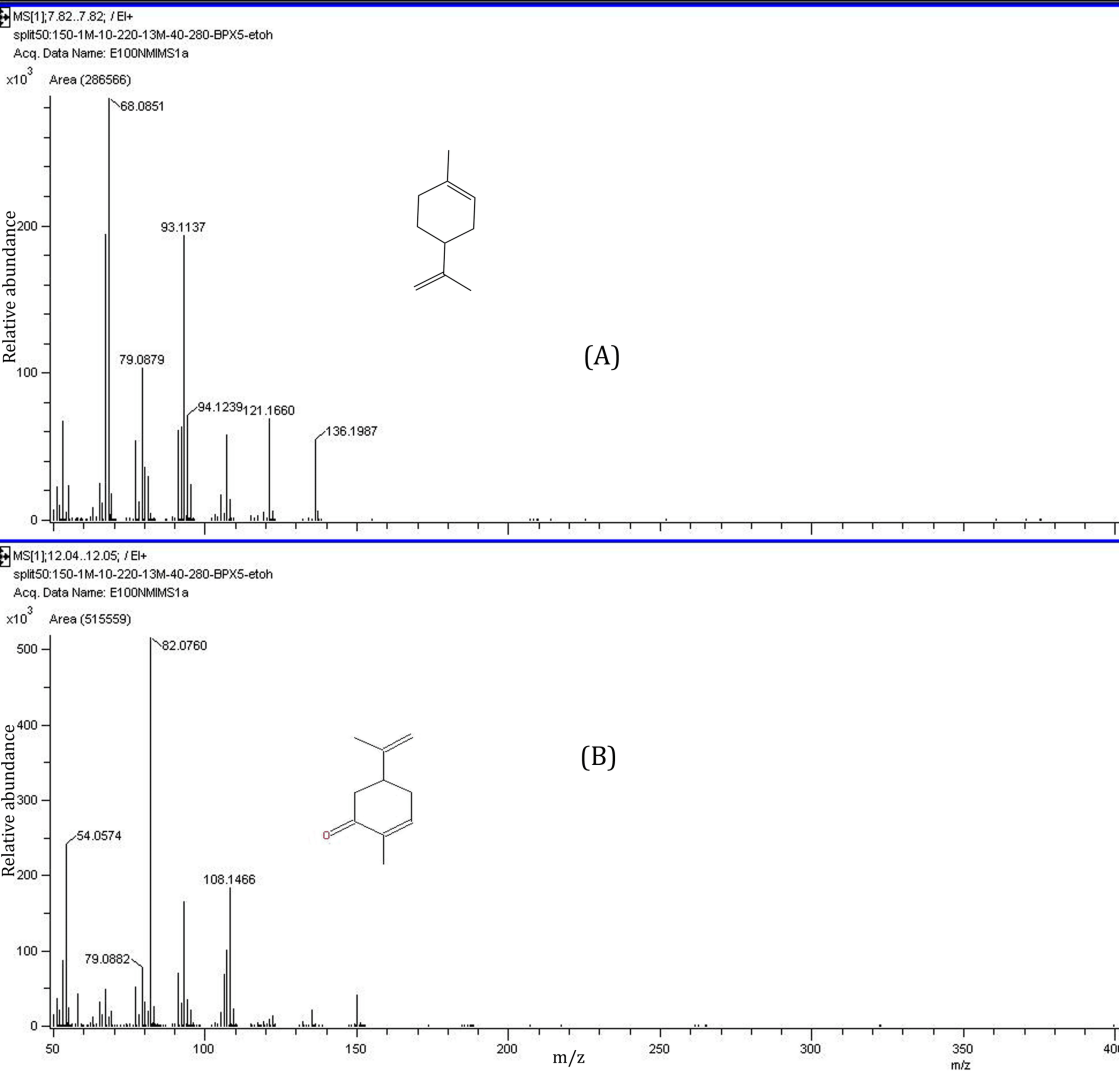
Mass spectrum of limonene (A) and carvone (B)

The chemical composition of the caraway oil was analyzed by GC-MS/flame ionization detector (FID). The identity of the main constituents was confirmed by their mass fragmentation analysis. The main compounds in oil were identified as limonene at a retention time of 7.81 minutes and carvone (55%) at a retention time of 12.04 minutes. (Figures 3, 4).

Behavioural assessment

Locomotor Activity

**Figure 5:**
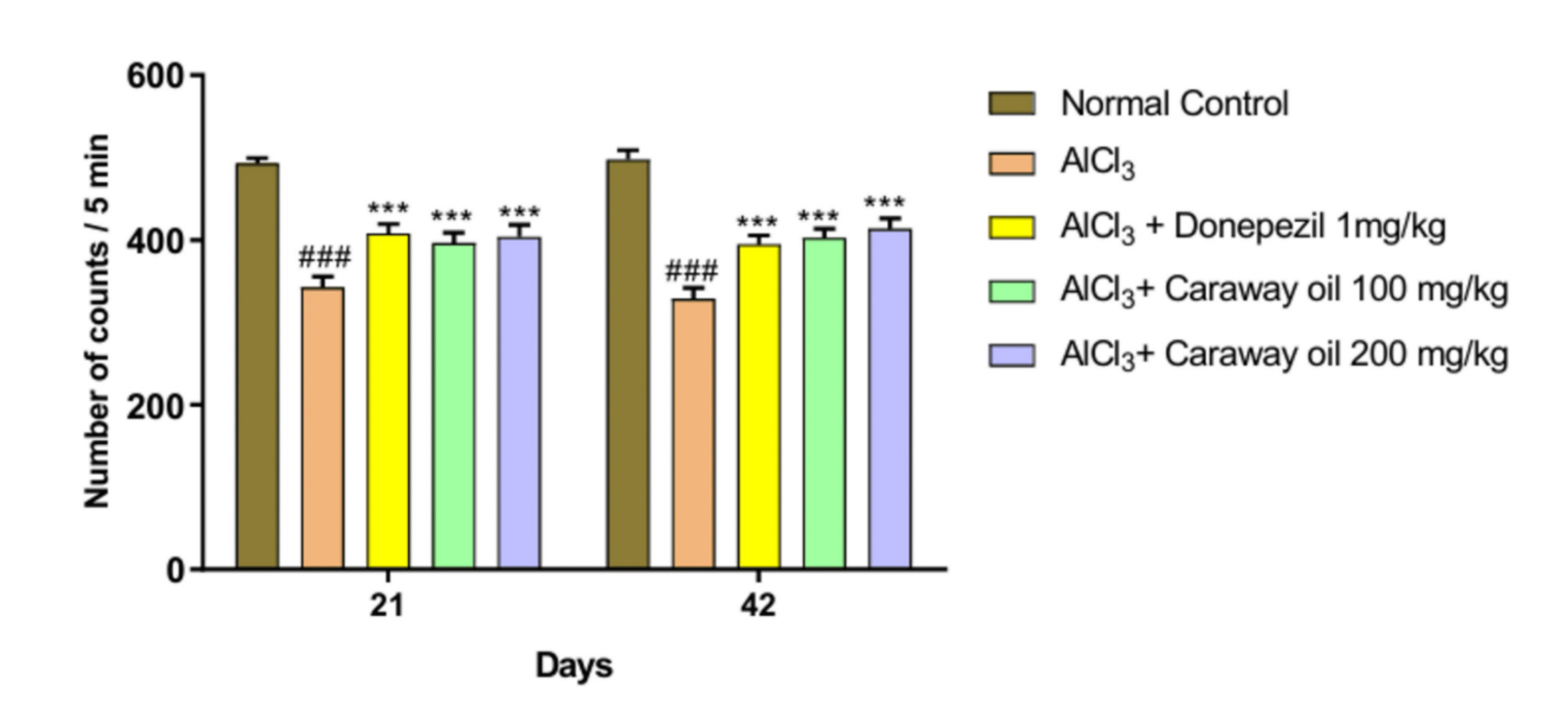
Effect of caraway oil on locomotor activity Data are expressed as mean ± SEM; ###p<0.001 when compared with normal control; ***p<0.001 when compared with the disease control group SEM: standard error of the mean; AlCl_3_: aluminum chloride

The aluminum chloride-treated group showed a significant decrease in the locomotor activity of animals compared to the normal control group. Caraway oil treatment at both selected dose levels significantly improved locomotor activity on days 21 and 42 (p < 0.001) when compared with the disease control group. Caraway oil treatment showed comparable results to those of the donepezil hydrochloride-treated group (Figure 5).

Morris Water Maze

**Figure 6:**
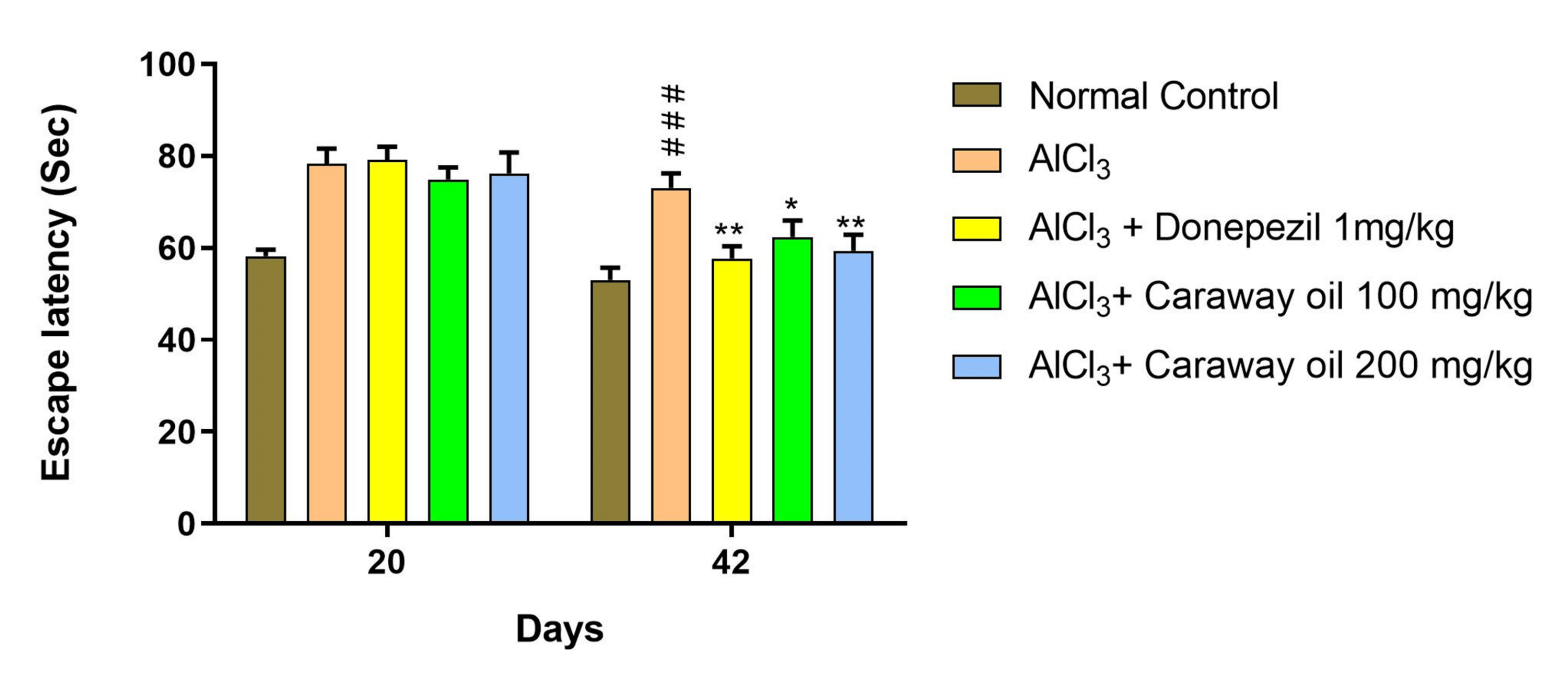
Effect of caraway oil on Morris water maze test Data are expressed as mean ± SEM (##p<0.01, ###p<0.001 when compared with normal control; *p<0.05, **p<0.01 when compared with the disease control group). SEM: standard error of the mean; AlCl_3_: aluminum chloride

The aluminum chloride-treated group showed a significant increase in escape latency compared to the normal control group. However, caraway oil treatment at a dose of 100 mg/kg (p < 0.05) and 200 mg/kg (p < 0.01) significantly prevented the increase in escape latency produced by aluminum chloride treatment on day 42 when compared with the disease control group. The caraway oil treatment showed comparable results to those of the donepezil hydrochloride-treated group and improved the retention performance of the spatial navigation task (Figure 6).

Elevated Plus Maze

**Figure 7:**
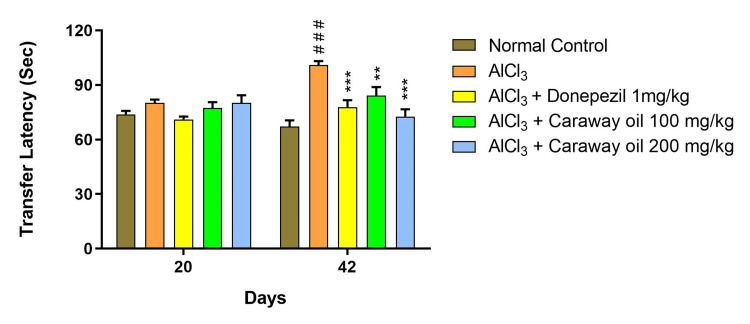
Effect of caraway oil on transfer latency Data are expressed as mean ± SEM (###p<0.001 when compared with normal control, *p<0.05, **p<0.01, ***p<0.001 when compared with the disease control group). SEM: standard error of the mean; AlCl_3_: aluminum chloride

The aluminum chloride-treated group showed an increase in first transfer latency and second transfer latency on days 21 and 42, respectively, with respect to initial transfer latency (ITL) at day 20, when compared with the normal control group. Caraway oil treatment at a dose of 200 mg/kg (p < 0.05) prevented an aluminum chloride-induced increase in first transfer latency on day 21 and second transfer latency on day 42 at a dose of 100 mg/kg (p < 0.01) and 200 mg/kg (p < 0.001) when compared with the disease control group. The caraway oil treatment showed comparable results to those of the donepezil hydrochloride-treated group on day 42, and it improved memory performance in animals (Figure 7).

Passive Avoidance Test

**Figure 8:**
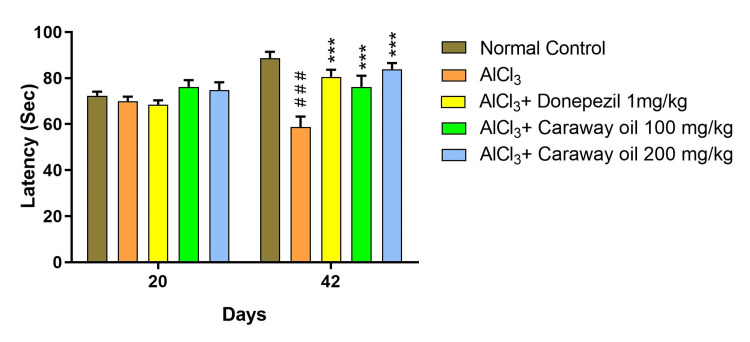
Effect of caraway oil on the latency Data are expressed as mean ± SEM (###p<0.001 when compared with normal control, *p<0.05, **p<0.01, ***p<0.001 when compared with the disease control group). SEM: standard error of the mean; AlCl_3_: aluminum chloride

In the passive avoidance test, the pre-shock latency was measured on day 20, and the post-shock latency was measured on days 21 and 42. In the aluminum chloride-treated group, a decrease in post-shock latency was reported as compared to the normal control group. Treatment with caraway oil at a dose of 200 mg/kg (p<0.01) showed significant recovery in post-shock latency on day 21 and at both dose levels of caraway oil on day 42 (p<0.001) when compared with the disease control group. Treatment with caraway oil showed comparable results to those of donepezil hydrochloride at a dose of 1 mg/kg, and the level of significance was found to be similar on day 42 (p<0.001) (Figure 8).

Assessment of oxidative stress parameters

Assessment of Oxidative Stress Parameters in the Hippocampus and Cortex

Aluminum chloride-treated animals showed an increase in malondialdehyde (MDA) level (p < 0.001) and a decrease in the level of glutathione (GSH) (p < 0.001), superoxide dismutase (SOD) (p < 0.001), and catalase activity (p < 0.001) in the hippocampus when compared with normal control animals. MDA level was reduced in caraway oil treatment at a dose of 100 mg/kg (p < 0.05) and 200 mg/kg (p < 0.01) when compared with the disease control group. Caraway oil treatment showed improvement in GSH and SOD levels at a dose of 200 mg/kg (p < 0.01) as compared with the disease control group. Treatment with caraway oil improved catalase activity at a dose of 100 mg/kg (p < 0.05) and 200 mg/kg (p < 0.01) as compared with the disease control group (Table 3).

**Table 3:** Effect of caraway oil on brain oxidative stress parameters in the hippocampus Data are expressed as mean ± SEM (###*p*<0.001 when compared with normal control, *p<0.05, **p<0.01, ***p<0.001 when compared with the disease control group). MDA: malondialdehyde; GSH: glutathione; SOD: superoxide dismutase; CAT: catalase activity; SEM: standard error of the mean; AlCl_3_: aluminum chloride

Sr No	Group	MDA	GSH	SOD	CAT
1	Normal control	3.217±0.2988	7.372±0.4751	6.187±0.3754	0.0071±0.00059
2	AlCl_3_	4.963±0.2478^###^	4.545±0.2998^###^	3.667±0.2871^###^	0.0033±0.00027^###^
3	AlCl_3_+Donepezil (1 mg/kg)	3.647±0.2665**	6.657±0.4068**	5.677±0.3734**	0.0058±0.0005**
4	AlCl_3_+Caraway oil (100 mg/kg)	3.968±0.2332*	5.518±0.4325	4.928±0.3608	0.0050±0.00029*
5	AlCl_3_+Caraway oil (200 mg/kg)	3.635±0.2305**	6.705±0.4693**	5.76±0.3788**	0.0057±0.00040**

The cortex region showed an increase in MDA level (p < 0.001) and a decrease in the level of GSH (p < 0.001), SOD (p < 0.001), and catalase activity (p < 0.001) in aluminum chloride-treated animals when compared with the normal control. Treatment with caraway oil reduced MDA level at a dose of 100 mg/kg (p < 0.05) and 200 mg/kg (p < 0.01) when compared with the disease control group. GSH and SOD levels were improved in caraway oil-treated animals at a dose of 200 mg/kg (p < 0.01) when compared with the disease control group. Catalase activity was improved in caraway oil treatment at a dose of 100 mg/kg (p < 0.05) and 200 mg/kg (p < 0.001) when compared with the disease control group. The caraway oil treatment showed comparable results to those of the donepezil hydrochloride-treated group at a dose of 1 mg/kg (Table 4).

**Table 4:** Effect of caraway oil on brain oxidative stress parameters in the cortex Data are expressed as mean ± SEM (###*p*<0.001 when compared with normal control, *p<0.05, **p<0.01, ***p<0.001 when compared with the disease control group). MDA: malondialdehyde; GSH: glutathione; SOD: superoxide dismutase; CAT: catalase activity; SEM: standard error of the mean; AlCl_3_: aluminum chloride

Sr No	Group	MDA	GSH	SOD	CAT
1	Normal control	3.753±0.2873	6.512±0.4161	6.272±0.3833	0.0073±0.000617
2	AlCl_3_	5.728±0.422^###^	4.132±0.3854^###^	3.42±0.4029###	0.003067±0.000341^###^
3	AlCl_3_+Donepezil (1 mg/kg)	4.262±0.3221**	5.685±0.3181**	4.972±0.2657**	0.005517±0.000467**
4	AlCl_3_+Caraway oil (100 mg/kg)	4.547±0.2155*	4.857±0.1777	4.338±0.3278	0.004933±0.000395*
5	AlCl_3_+Caraway oil (200 mg/kg)	4.167±0.2138**	5.975±0.1806**	5.263±0.1981**	0.005983±0.000494***

AChE

Aluminum chloride-treated animals showed a significant increase in AChE activity (p < 0.001) in the hippocampus region when compared with normal control animals. Treatment with caraway oil significantly decreased AChE activity at a dose of 100 mg/kg (p < 0.05) and 200 mg/kg (p < 0.01) when compared with disease control animals. The caraway oil treatment showed comparable results to those of the donepezil hydrochloride-treated group (Figure 09). The cortex region showed a significant increase in AChE activity (p < 0.001) in aluminum chloride-treated animals when compared with normal control animals. Caraway oil treatment significantly decreased AChE activity at a dose of 100 mg/kg (p < 0.05) and 200 mg/kg (p < 0.001) when compared with the disease control (Figure 10).

**Figure 9:**
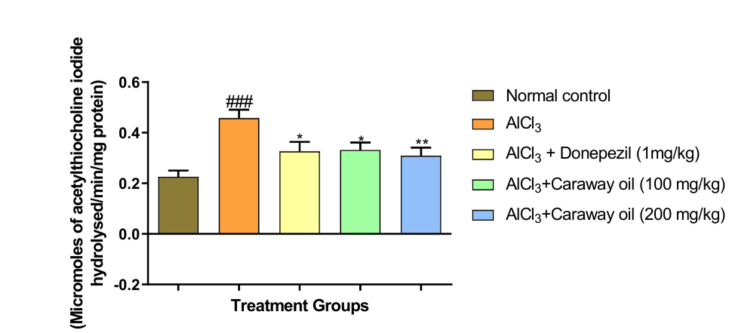
Effect of caraway oil on acetylcholinesterase assay in hippocampus Data are expressed as mean ± SEM (###*p *< 0.001 when compared with normal control, ***p< 0.001, **p< 0.01, *p< 0.05 when compared with the disease control group). SEM: standard error of the mean; AlCl_3_: aluminum chloride

**Figure 10:**
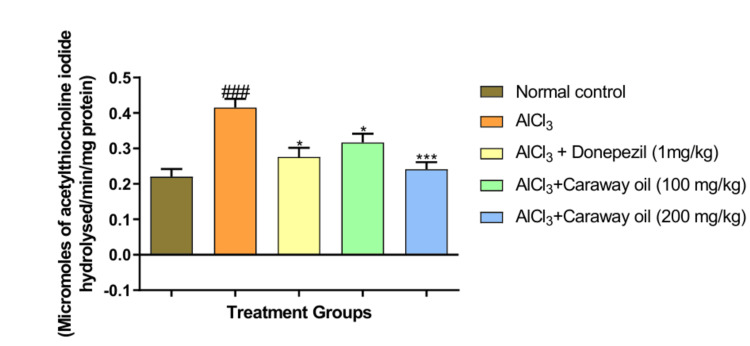
Effect of caraway oil on acetylcholinesterase assay in cortex Data are expressed as mean ± SEM (###*p*< 0.001 when compared with normal control, ***p< 0.001, *p< 0.05 when compared with the disease control group). SEM: standard error of the mean; AlCl_3_: aluminum chloride

## Discussion

Treatment with caraway oil showed significant improvement in behavioral parameters, oxidative stress parameters, and inhibition of AChE activity in aluminum chloride-induced neurotoxicity. GC-MS is used to analyze essential oils because it efficiently separates and isolates the volatile components, allowing for thorough chemical characterization and quality evaluation. GC separates the components of the essential oil according to volatility, whereas MS recognizes them by their distinct mass-to-charge ratios [26]. In this study, caraway oil was analyzed through GC-MS, and it was observed that carvone (55%) and limonene were major components in caraway oil. The study reported that carvone and limonene improve cognition and oxidative stress in neurodegenerative disease [27]. The treatment with carvone-rich caraway oil can be useful as an alternative treatment for AD. Aluminum chloride, a neurotoxin, produces reactive oxygen species, which can cause oxidative stress in the brain. Reactive oxygen species are toxic byproducts of regular cellular metabolism that have the potential to destroy tissues and cells. The development of certain pathological alterations associated with AD, including the development of amyloid plaques and NFTs, is influenced by oxidative stress, a known risk factor for the disease [28]. Aluminum chloride can disrupt the brain's antioxidant defense system and increase reactive oxygen species production, leading to damage to DNA, proteins, and lipids [29].

Aluminum chloride administration showed cognitive and behavioral deficits, as evidenced by reduced locomotor activity, impaired spatial memory, increased anxiety-like behavior, and deficits in learning and memory. Caraway oil administration (both 100 mg/kg and 200 mg/kg) significantly ameliorated these behavioral deficits. Improvements were observed in locomotor activity, spatial learning, and memory retention, with efficacy comparable to the standard treatment, donepezil. These findings suggest that caraway oil can counteract aluminum chloride-induced neurotoxicity and restore cognitive function.

Caraway oil significantly attenuated aluminum chloride-induced oxidative stress in the hippocampus and cortex, as evidenced by malondialdehyde, a marker of lipid peroxidation, increased superoxide dismutase and catalase activities, and restoration of glutathione levels. These effects underscore the potent antioxidant properties of caraway oil’s constituents, which likely mitigate the oxidative stress central to pathogenesis. Carvone has been reported to have an antioxidant effect, which is responsible for a reduction in oxidative damage [30].

Acetylcholine (ACh) is a cholinergic neurotransmitter that mediates learning and memory, along with other basic functions. ACh is hydrolyzed into choline and acetate by the enzyme AChE. AD is linked to ACh deficit, and cognitive function is enhanced by decreasing AChE activity to increase ACh levels [31]. AChE activity, which is typically elevated in AD and contributes to cholinergic deficit, was significantly reduced by caraway oil treatment. This suggests that caraway oil can enhance cholinergic neurotransmission, a key therapeutic approach in AD. The present study provides scientific support for the use of caraway oil in aluminum chloride-induced neurotoxicity in rats. The major limitation of the research work is the lack of expression study of an important protein such as amyloid beta in specific regions of the brain. 

## Conclusions

Treatment with caraway oil improved memory and antioxidant enzymes in animals. The treatment decreased AChE activity in the cortex and hippocampus. Through oxidative stress management and AChE activity reduction, caraway oil demonstrated a neuroprotective effect, which exhibits potential benefits of caraway oil in the management of AD. Further, clinical studies may be conducted to establish the effect of caraway oil in AD. 

## References

[1] Atri A (2019). The Alzheimer’s disease clinical spectrum: diagnosis and management. Med Clin North Am.

[2] Jack CR Jr, Knopman DS, Jagust WJ (2013). Tracking pathophysiological processes in Alzheimer's disease: an updated hypothetical model of dynamic biomarkers. Lancet Neurol.

[3] Tahami Monfared AA, Byrnes MJ, White LA, Zhang Q (2022). Alzheimer's disease: epidemiology and clinical progression. Neurol Ther.

[4] Knopman DS, Amieva H, Petersen RC (2021). Alzheimer disease. Nat Rev Dis Primers.

[5] Maya S, Prakash T, Madhu KD, Goli D (2016). Multifaceted effects of aluminium in neurodegenerative diseases: a review. Biomed Pharmacother.

[6] Kaizer RR, Corrêa MC, Spanevello RM, Morsch VM, Mazzanti CM, Gonçalves JF, Schetinger MR (2005). Acetylcholinesterase activation and enhanced lipid peroxidation after long-term exposure to low levels of aluminum on different mouse brain regions. J Inorg Biochem.

[7] Andrieu S, Coley N, Lovestone S, Aisen PS, Vellas B (2015). Prevention of sporadic Alzheimer's disease: lessons learned from clinical trials and future directions. Lancet Neurol.

[8] Passeri E, Elkhoury K, Morsink M (2022). Alzheimer’s disease: treatment strategies and their limitations. Int J Mol Sci.

[9] Abd Rashed A, Abd Rahman AZ, Rathi DN (2021). Essential oils as a potential neuroprotective remedy for age-related neurodegenerative diseases: a review. Molecules.

[10] Johri RK (2011). Cuminum cyminum and Carum carvi: an update. Pharmacogn Rev.

[11] Owokotomo IA, Ekundayo O, Abayomi TG, Chukwuka AV (2015). In-vitro anti-cholinesterase activity of essential oil from four tropical medicinal plants. Toxicol Rep.

[12] Sharma N, Tan MA, An SS (2021). Mechanistic aspects of Apiaceae family spices in ameliorating Alzheimer’s disease. Antioxidants (Basel).

[13] Sadowska U, Matwijczuk A, Niemczynowicz A, Dróżdż T, Żabiński A (2019). Spectroscopic examination and chemometric analysis of essential oils obtained from Peppermint herb (Mentha piperita L.) and caraway fruit (Carum carvi L.) subjected to pulsed electric fields. Processes.

[14] Cerceau CI, Barbosa LC, Alvarenga ES, Ferreira AG, Thomasi SS (2016). A validated (1)H NMR method for quantitative analysis of α-bisabolol in essential oils of Eremanthus erythropappus. Talanta.

[15] Adams RP (2007). Identification of Essential Oil Components by Gas Chromatography/Quadrupole Mass Spectroscopy. 4th Edition. https://www.researchgate.net/publication/283650275_Identification_of_Essential_Oil_Components_by_Gas_ChromatographyQuadrupole_Mass_Spectroscopy.

[16] Kumar A, Dogra S, Prakash A (2009). Protective effect of curcumin (Curcuma longa), against aluminium toxicity: possible behavioral and biochemical alterations in rats. Behav Brain Res.

[17] Morris R (1984). Developments of a water-maze procedure for studying spatial learning in the rat. J Neurosci Methods.

[18] Rao Barkur R, Bairy LK (2015). Evaluation of passive avoidance learning and spatial memory in rats exposed to low levels of lead during specific periods of early brain development. Int J Occup Med Environ Health.

[19] Sharma AC, Kulkarni SK (1992). Evaluation of learning and memory mechanisms employing elevated plus-maze in rats and mice. Prog Neuropsychopharmacol Biol Psychiatry.

[20] Auti ST, Kulkarni YA (2019). Neuroprotective effect of cardamom oil against aluminum induced neurotoxicity in rats. Front Neurol.

[21] Ellman GL, Courtney KD, Andres V, Featherstone RM (1961). A new and rapid colorimetric determination of acetylcholinesterase activity. Biochem Pharmacol.

[22] Ohkawa H, Ohishi N, Yagi K (1979). Assay for lipid peroxides in animal tissues by thiobarbituric acid reaction. Anal Biochem.

[23] Paoletti F, Aldinucci D, Mocali A, Caparrini A (1986). A sensitive spectrophotometric method for the determination of superoxide dismutase activity in tissue extracts. Anal Biochem.

[24] Lück H (1965). Catalase. Methods of Enzymatic Analysis (Second Printing, Revised).

[25] Ellman GL (1959). Tissue sulfhydryl groups. Arch Biochem Biophys.

[26] Marriot PJ, Shellie R, Cornwell C (2001). Gas chromatographic technologies for the analysis of essential oils. J Chromatogr A.

[27] Wojtunik-Kulesza KA, Rudkowska M, Klimek K, Mołdoch J, Agacka-Mołdoch M, Budzyńska B, Oniszczuk A (2024). S-(+)-carvone, a monoterpene with potential anti-neurodegenerative activity—in vitro, in vivo and ex vivo studies. Molecules.

[28] Promyo K, Iqbal F, Chaidee N, Chetsawang B (2020). Aluminum chloride-induced amyloid β accumulation and endoplasmic reticulum stress in rat brain are averted by melatonin. Food Chem Toxicol.

[29] Rui D, Yongjian Y (2010). Aluminum chloride induced oxidative damage on cells derived from hippocampus and cortex of ICR mice. Brain Res.

[30] Pina LT, Serafini MR, Oliveira MA, Sampaio LA, Guimarães JO, Guimarães AG (2022). Carvone and its pharmacological activities: a systematic review. Phytochemistry.

[31] Agrawal M, Singhal M, Semwal BC (2024). Neuroprotective action of hordenine against the aluminium chloride (AlCl3) induced Alzheimer’s diseases & associated memory impairment in experimental rats. Pharmacol Res - Mod Chin Med.

